# Correlation analysis of fundamental movement skills level and aquatic competence acquisition in children aged 4–6 years

**DOI:** 10.3389/fpubh.2026.1706377

**Published:** 2026-02-04

**Authors:** Hai-Yan Li, Guo-Hui Zhao

**Affiliations:** 1 School of Physical Education, Shanxi University, Taiyuan, Shanxi, China; 2 School of Sport Science, Beijing Sport University, Beijing, China

**Keywords:** acquisition, aquatic competence, correlation analysis, fundamental movement skills, preschool children

## Abstract

**Purpose:**

This study aims to explore the correlation between Fundamental Movement Skills (FMS) and Aquatic Competence (AC) acquisition in preschool children aged 4–6 years.

**Methods:**

A total of 120 preschool children aged 4–6 years with no swimming learning experience (60 boys and 60 girls) were recruited in Beijing. All subjects were stratified and randomly divided into an experimental group and a control group (30 boys and 30 girls). Before and after a 6-week (twice a week, total of 12 sessions) aquatic movement skills course in the experimental group, all children underwent FMS and AC testing. Descriptive statistics were performed on the results of the two tests, and the Mann–Whitney U test was used to compare the ΔAC of the two groups of subjects. Analysis of covariance (ANCOVA) and rank-transformed ANCOVA were used for inter-group difference analysis. Spearman rank correlation analysis was used to explore the correlation between FMS and AC acquisition.

**Results:**

The locomotor skills, object control, and FMS scores of the experimental group and the control group in the post-test were all higher than those in the pre-test, and the AC post-test score of the experimental group was higher than the pre-test. The improvement levels of locomotor skills (*F* = 18.98, *p* < 0.01, η^2^ = 0.14), object control (*F* = 32.19, *p* < 0.01, η^2^ = 0.22), FMS (*F* = 45.85, *p* < 0.01, η^2^ = 0.28), and AC (*F* = 1663.96, *p* < 0.01, η^2^ = 0.93) in the experimental group were significantly better than those in the control group. At the same time, the improvement level of Aquatic Competence (ΔAC) in the experimental group was significantly higher than that of the control group (*Z* = −9.48, *p* < 0.01). Correlation analysis indicates that the acquisition of AC in boys is significantly positively correlated with object control skills (*r* = 0.419, *p* = 0.021) and FMS (*r* = 0.388, *p* = 0.034).

**Conclusion:**

Learning aquatic movement skills can significantly enhance the fundamental movement skills and aquatic competence of preschool children aged 4–6 years. The control skills of boys are related to their aquatic competence acquisition. Therefore, during the preschool stage, focusing on and cultivating children’s fundamental movement skills, especially object control abilities, is of great significance for their effective development of future aquatic competence.

## Introduction

The preschool stage is a critical window for the development of children’s Fundamental Movement Skills (FMS). During this period, children’s brain neuroplasticity is strong, and their movement patterns are evolving from initial clumsy attempts to skilled and automated actions, laying the foundation for lifelong participation in physical activities and healthy living (1). FMS is a set of foundational movement abilities that form the basis for more complex and specialized motor skills, typically divided into three categories: locomotor skills, such as running, jumping, sliding, and hopping, which enable individuals to move in space; object control skills, such as throwing, catching, kicking, and striking, which involve the ability to manipulate objects; and stability skills, such as turning, balancing, and shifting center of gravity, which pertain to the control of body posture (2).

A substantial body of strong longitudinal research indicates that the proficiency of FMS is a key indicator for predicting children’s current and future overall health, with impacts spanning multiple dimensions (3, 4). In terms of physical health, FMS proficiency is significantly positively correlated with higher levels of daily moderate-to-vigorous physical activity (MVPA), serving as an important protective factor against childhood obesity and its related metabolic diseases, such as Type 2 diabetes (5, 6). Conversely, children with delayed FMS development are more likely to fall into a vicious cycle of “insufficient skill proficiency-frustration in sports participation-decreased willingness to be active-reduced opportunities for skill development.” In the cognitive and socio-emotional domains, the impact is equally profound. Children who demonstrate advantages in executive function tasks often also possess better FMS levels (7), suggesting that there may be shared neural mechanisms underlying both. Additionally, successfully applying motor skills in group games (such as accurately passing the ball to a teammate) can effectively enhance children’s self-efficacy, self-esteem, and promote peer acceptance and social interaction, positively impacting their social development (8–10).

At the same time, aquatic competence as a crucial motor skill in a specific environment (11, 12), has also received widespread attention in recent years. The unique sensory inputs provided by the water environment (such as buoyancy and resistance) offer children different movement challenges and learning opportunities compared to land (13, 14). Mastering aquatic competence is not only a core skill related to life safety, significantly reducing the risk of drowning (which remains one of the leading causes of accidental death among children globally), but it has also been shown to have diverse benefits for children’s development (15–17). Regular participation in structured water activities can effectively promote children’s cardiopulmonary endurance, muscle strength, flexibility, and unique aquatic balance and coordination skills (18, 19). More importantly, overcoming initial fears of water, learning to float and move, can greatly enhance children’s confidence, risk perception abilities, and resilience (20), and these positive psychological benefits often transfer to other areas of life. Although FMS and aquatic competence differ in their specific manifestations and environments, there may be shared neuromuscular control mechanisms and sensory integration processes in aspects such as motor learning and control, and body coordination and perception. For example, the bilateral coordination, timing of force application, and body posture control required in locomotor skills (such as double-leg jumps and single-leg hops) share structural similarities with the force patterns in water push-offs or frog kick movements. Therefore, we can theoretically infer that children who develop FMS better on land may possess superior adaptability in their nervous systems and bodies, enabling them to learn, adapt, and master the complex new skills required in aquatic environments more effectively. However, current research often isolates the developmental value of each, and empirical studies on the intrinsic connections between them, especially in the rapidly developing preschool child population, are still insufficient and lack depth. Existing limited evidence is mostly focused on adolescents or adults, or only provides simple phenomenological descriptions without delving into potential mechanisms of connection.

Based on the theoretical background and research gaps mentioned above, this study aims to systematically explore the correlation between FMS levels and the acquisition of aquatic competence in preschool children aged 4–6 years. This study proposes the following specific hypotheses: (1) The FMS levels of preschool children are positively correlated with their acquisition of aquatic competence; (2) This correlation may exhibit different intensity patterns across different genders. The results of this study are expected to provide important empirical evidence for understanding the transfer effects between fundamental movement skills and aquatic competence, and to offer key theoretical foundations and practical guidance for the future development of integrated intervention programs that combine land and aquatic training aimed at comprehensively enhancing children’s physical literacy and safety skills.

## Study methods

### Participants

This study recruited 120 preschool children aged 4–6 years old in Beijing (60 boys and 60 girls) with no prior swimming experience. The subjects were stratified by gender and randomly divided into an experimental group and a control group, with 60 participants in each group (30 boys and 30 girls). All children had no significant diseases or physical disabilities that would affect their participation in sports. The guardians of the participants voluntarily signed an informed consent form, understanding the purpose, process, and risks of the study, and agreed to participate in the research. Ethical approval was obtained from the Ethics Committee of Shanxi University (Approval No: SXULL2024116).

### Randomization methodology

This study employed a two-arm, assessor-blinded, stratified randomized controlled trial design. An independent researcher not involved in subsequent recruitment, intervention, or data collection generated the randomization sequence using IBM SPSS Statistics software (version 26.0). To ensure gender balance between groups, randomization was stratified by gender (male and female strata). Within each gender stratum, blocked randomization with a fixed block size of 4 was applied to produce a sequence allocating participants to either the experimental or control group. To strictly maintain allocation concealment and prevent selection bias, the generated allocation sequence was concealed in sequentially numbered, opaque sealed envelopes. Each envelope was labeled only with the sequence number and gender stratum. After participants completed baseline assessments and were formally enrolled, the intervention coordinator opened the next corresponding envelope in the sequence for the appropriate gender stratum and informed the participant of their group assignment according to the allocation card inside. Recruitment, baseline assessment, and randomization were performed by different personnel to protect blinding. Outcome assessors remained blinded to group assignment throughout the trial. Due to the nature of the intervention (aquatic exercise training), blinding of participants and intervention coaches was not feasible.

### Fundamental movement skills

The second edition of the Test of Gross Motor Development (TGMD-2) is used to evaluate the FMS level of preschool children. The TGMD-2 test includes two subcategories: locomotor skills and object control skills. The locomotor skills test includes six actions: running, sliding forward, hopping on one foot, jumping forward, standing long jump, and sliding sideways; the object control skills include six actions: striking a stationary ball, dribbling in place, catching, kicking, overhand throwing, and rolling a ball, totaling 12 test items (21). Each action contains 3 to 5 levels, with a maximum score of 3 to 5 points. The FMS score is the sum of the scores for each movement. Before the test, all testers and scorers evaluates received unified training from experts in early childhood motor development, and they are proficient in the testing process and scoring criteria. During the test, the tester provides verbal guidance and demonstrations, and the subject practices once before undergoing two rounds of formal testing. The entire testing process is recorded using a camera, and the scorer evaluates based on the video. If two raters score a certain action inconsistently, a third rater will score it and ultimately reach an agreement. The intraclass correlation coefficient (ICC) within this research group is 0.99.

### Aquatic competence

This study selects the Actual Aquatic Skills Test (AAST) to assess the aquatic abilities of the participating children (22). The AAST includes 17 technical actions across 9 skills: entering the water, breathing control, floating, underwater orientation, propulsion, submersion, exiting the water, gliding, and visual skills. All 17 technical actions are presented with illustrations. Each technical action is constructed with three levels: Level 1 = “unable to complete the skill”; Level 2 = “skill in progress,” meaning the quality of completion is low or requires assistance from equipment; Level 3 = “able to complete the skill.” The AC score is the sum of the scores for each movement” in the methods section. The reliability and validity of the scale were verified through testing by children and an expert panel (23).

During the test, a coach with extensive experience in teaching swimming to preschool children demonstrates Level 3 actions in the water. If the child cannot complete it after two attempts, the coach demonstrates Level 2 actions. If the child still cannot complete it after two attempts, the testing for that action ends. Meanwhile, two researchers on the shore (prior to the test, the two researchers received standardized training from experts in preschool children’s motor development, and they were proficient in the scoring criteria) scored based on the quality of the child’s movements; if there is inconsistency, they communicate to unify or further review the video to provide a final result. The intra-class correlation coefficient (ICC) for this research group is 0.98. Throughout the entire testing process, there was a lifeguard responsible for child safety and possessing relevant first aid qualifications for protection.

### Experimental procedure

First, all subjects underwent FMS and AC testing; then, the experimental group received a 6-week (2 times a week, a total of 12 times) intervention of aquatic movement skills course learning, while the control group maintained normal conditions; finally, all subjects were retested for FMS and AC.

The intervention measures for the experimental group included: the course content mainly consisted of basic aquatic movement skills, such as floating, breathing control, kicking, arm strokes, and water orientation. Each class lasted 45 min, including warm-up, review, new skill learning, and relaxation activities. Small-class teaching was adopted, with 10 children per class, ensuring that each child received adequate attention and guidance, and the course content was uniform for all children. Classes were taught by professionally qualified swimming coaches who had received training in child psychology, motor skill development, and other related areas. Meanwhile, the control group maintained their daily activities without additional aquatic movement skills training during the intervention period.

### Statistical analysis

All raw data were entered and cross-checked to ensure accuracy. The normality of variables was assessed using the Shapiro–Wilk test. Variables conforming to a normal distribution are described using mean ± standard deviation (X ± S), while those not conforming to a normal distribution are described using median (P25, P75). Descriptive statistics were performed on the results of the two tests, and the Mann–Whitney U test was used to compare the ΔAC of the two groups of subjects. Analysis of covariance (ANCOVA) and rank-transformed ANCOVA were used to analyze between-group differences for normally and non-normally distributed data, respectively, with group as a fixed factor and age, gender, and corresponding baseline scores included as covariates. The correlation between fundamental motor skills and aquatic skill acquisition was analyzed using Spearman’s rank correlation. All statistical analyses were performed using SPSS 26.0, with the significance level set at α = 0.05.

## Results

The research results show that there are no significant differences in age, height, weight, and BMI between the experimental group and the control group (*p* > 0.05), which reduces the impact of individual differences on the research results. Basic information of the subjects is shown in Table 1.

**Table 1:** Basic information of subjects.

Group	Total (*N* = 120)	Age	Height (cm)	Weight (kg)	BMI (kg/m^2^)
Experimental group	30 Boys	5.0 ± 0.7	111.9 ± 8.2	20.6 ± 6.3	16.5 ± 5.6
	30 Girls	5.2 ± 0.9	112.5 ± 7.0	21.0 ± 6.8	16.0 ± 5.8
Control group	30 Boys	4.9 ± 0.8	110.0 ± 7.7	20.9 ± 5.8	17.3 ± 5.2
	30 Girls	5.1 ± 1.0	112.6 ± 9.8	21.1 ± 5.1	16.6 ± 5.1

Through the pre-test and post-test, it was found that the locomotor skills, object control, and FMS scores of both the experimental group and the control group were higher in the post-test than in the pre-test. The post-test score of the experimental group’s AC was higher than the pre-test (see Table 2). By controlling the effects of baseline scores, age, and gender through ANCOVA and rank transformation ANCOVA, it was found that the improvement levels of locomotor skills (*F* = 18.98, *p* < 0.01, η^2^ = 0.14), object control (*F* = 32.19, *p* < 0.01, η^2^ = 0.22), FMS (*F* = 45.85, *p* < 0.01, η^2^ = 0.28), and AC (*F* = 1663.96, *p* < 0.01, η^2^ = 0.93) in the experimental group were significantly better than those in the control group. In further comparisons of different genders among children, it was found that the improvement levels of all indicators in the experimental group were also better than those in the control group (*p* < 0.01). The improvement level of swimming ability (*Δ* swimming ability) in the experimental group was significantly higher than that in the control group (*Z* = −9.48, *p* < 0.01), and this was true for both boys (*Z* = −6.68, *p* < 0.01) and girls (*Z* = −6.69, *p* < 0.01). This result emphasizes the effectiveness of the intervention in improving aquatic ability. However, no gender differences were observed in the pre-test and post-test for locomotor skills, object control skills, FMS, aquatic ability, and *Δ* aquatic ability between the experimental and control groups (*p* > 0.05). The specific results are shown in Table 3.

**Table 2:** Test results.

Indicator	Population	Experimental group		Control group	
		Pre-test	Post-test	Pre-test	Post-test
Locomotor	Overall	36.0 (32.0, 42.0)	41.0 (38.0, 47.0)	37.0 (31.0, 39.0)	39.7 ± 6.4
	Boys	39.0 (33.0, 44.0)	43.0 (38.0, 48.0)	36.0 ± 5.0	40.0 ± 4.3
	Girls	35.0 (32.0, 41.0)	39.0 (38.0, 48.0)	36.6 ± 7.1	40.0 (35.0, 42.0)
Object control	Overall	29.0 (27.0, 32.0)	34.4 ± 5.3	30.7 ± 4.9	31.0 (30.0, 34.0)
	Boys	30.0 (28.0, 32.0)	34.6 ± 4.8	30.0 ± 4.6	31.0 (30.0, 34.0)
	Girls	28.5 ± 4.3	35.0 (32.8, 37.0)	31.1 ± 5.1	32.3 ± 4.1
FMS	Overall	65.0 (59.0, 75.0)	76.0 (70.0, 82.0)	67.0 (61.0, 69.0)	72.0 (66.0, 75.0)
	Boys	68.0 (62.0, 76.0)	77.0 (70.0, 83.0)	67.0 (62.0, 69.0)	73.0 (67.0, 75.0)
	Girls	64.0 (59.0, 73.0)	75.5 ± 6.4	67.7 ± 10.9	72.1 ± 9.0
AC	Overall	21.0 (20.0, 22.0)	49.0 (43.0, 50.0)	22.0 (20.0, 22.0)	21.0 (21.0, 22.0)
	Boys	21.0 (20.0, 21.0)	49.0 (43.0, 52.0)	22.0 (20.0, 22.0)	22.6 ± 1.3
	Girls	20.4 ± 1.8	46.0 (43.0, 50.0)	22.0 (19.0, 22.0)	22.0 (21.0, 23.0)
ΔAC	Overall	28.0 (23.0, 30.0)		0 (−1.0, 2.0)	
	Boys	29.0 (23.0, 31.0)		0.0 (−1.0, 1.0)	
	Girls	25.0 (23.0, 30.0)		0.5 (−1.25, 2.0)	

**Table 3:** Comparison of test results between groups.

Indicator	Population	*F*	η^2^	*P*	*Z*
Locomotor	Overall	18.98	0.14	<0.01	
	Boys	9.02	0.14	<0.01	
	Girls	10.15	0.15	<0.01	
Object control	Overall	32.19	0.22	<0.01	
	Boys	28.97	0.34	<0.01	
	Girls	7.82	0.12	<0.01	
FMS	Overall	45.85	0.28	<0.01	
	Boys	35.01	0.38	<0.01	
	Girls	14.32	0.2	<0.01	
AC	Overall	1663.96	0.93	<0.01	
	Boys	950.07	0.94	<0.01	
	Girls	722.83	0.93	<0.01	
ΔAC	Overall			<0.01	−9.48
	Boys			<0.01	−6.68
	Girls			<0.01	−6.69

Correlation analysis between FMS and AC: Before the intervention, there was no significant correlation between locomotor skills, object control, and FMS with AC; after the intervention, the improvement level of AC (ΔAC) showed a significant positive correlation with object control skills (boys, overall) (*r* = 0.419, *p* = 0.021; *r* = 0.280, *p* = 0.031). There is a significant positive correlation between FMS and ΔAC in boys (*r* = 0.388, *p* = 0.034). Meanwhile, there is a positive correlation trend between FMS and ΔAC (*r* = 0.224, *p* = 0.085), suggesting that FMS ability may also have a potential relationship with the improvement of aquatic Competence, especially evident in boys (see Table 4).

**Table 4:** Correlation analysis of FMS and AC.

Indicator	Population	AC Pre-test		ΔAC	
		Correlation coefficient	*P* value	Correlation coefficient	*P* value
Locomotor	Overall	−0.051	0.697	0.077	0.559
	Boys	−0.105	0.581	0.286	0.125
	Girls	−0.049	0.795	−0.021	0.911
Object control	Overall	−0.165	0.208	0.28	0.031^*^
	Boys	−0.268	0.153	0.419	0.021^*^
	Girls	−0.205	0.276	0.159	0.404
FMS	Overall	−0.14	0.286	0.224	0.085
	Boys	−0.176	0.353	0.388	0.034^*^
	Girls	−0.187	0.323	0.071	0.711

*Represents (*p* < 0.05).

## Discussion

This study aims to explore the correlation between fundamental movement skills and AC acquisition in preschool children aged 4–6 years. Through a 6-week interventional research involving 120 preschool children, we found that the experimental group made significant progress in FMS, locomotor skills, object control, and overall AC, with the improvement far exceeding that of the control group. More importantly, the study revealed a significant positive correlation between boy’s object control ability, FMS and their level of improvement in AC after the intervention, providing a new perspective for understanding the influencing factors of children’s aquatic competence development.

After implementing a 6-week aquatic motor skills learning course, twice a week, the experimental group demonstrated significant improvements in locomotor skills, motor control, total FMS score, and AC, significantly outperforming the control group. The research results strongly demonstrate that a systematic aquatic motor skills learning course can effectively promote the synchronized development of FMS and AC in preschool children (24, 25). Therefore, aquatic motor skills learning is not merely a simple aquatic activity; it does have a transfer effect on children’s physical coordination, balance, explosive power, and other basic movement patterns (19, 26). Through aquatic motor skills learning, children are compelled to repeatedly apply and reinforce basic movement patterns, thereby promoting an increase in FMS scores. Compared to the significant improvement in AC in the experimental group, the control group showed almost no change, indicating that a professional aquatic motor skills course is a direct and effective way to enhance children’s AC (27–29). The 17 aquatic skills measured in this study encompass various basic skills such as floating, underwater breathing, kicking, turning in water, and emerging from water, which require children to possess certain physical control abilities, adaptability to the water environment, and coordination of movements. The experimental group significantly outperformed the control group in the level of improvement in aquatic competence, further emphasizing the effectiveness of the intervention and ruling out the influence of pre-existing differences in ability within the sample on the results.

The study revealed the correlation between fundamental movement skills and the improvement level of AC. Although there was no significant correlation between locomotor skills, object control, and the total FMS score and the pre-test score of aquatic competence before the intervention, this may indicate that without specific training, children’s FMS abilities have not yet established a close connection with their basic adaptability in water (15). However, after the intervention, a significant positive correlation was observed between object control skills (boys, overall), FMS (boys), and the improvement level of AC. This result has very important theoretical and practical significance. Object control ability is an important component of FMS. In the aquatic environment, children need to have better body control abilities to cope with the buoyancy, resistance of water, and changes in their center of gravity (14, 26, 30). Higher body control ability means that children can use different parts of their bodies more coordinately, making movements smoother and more coherent. In water, this coordination is crucial for actions such as kicking, stroking, and body turning (31, 32). Better body control ability may also be related to children’s more acute perception of the water environment. When the body is subjected to thrust or resistance in water, children with good control ability can adjust their body posture more quickly to adapt to these changes, without stopping movement or experiencing fear due to loss of control. In water, good body control ability is the foundation of safety (33). It helps children maintain stability in unfamiliar water environments, reducing the risk of accidental choking or falling, thus encouraging them to try and learn aquatic skills (15, 18). Only the object control skills of boys and FMS show a significant positive correlation with their ΔAC, which may be related to their understanding of object motion trajectories, spatial positioning, and quick responses (34). This provides a more solid foundation for their “object manipulation” or “precise movement” underwater or in water-related activities, enabling them to more effectively transfer and apply these existing skills when learning aquatic movement skills.

The positive correlation between object control and ΔAC suggests that children with stronger object control abilities on land show more significant improvements in their aquatic competence after receiving training in water movement skills. Their bodies respond better to aquatic movement instructions, allowing them to master and execute new aquatic skills more effectively. This may be reflected in the fact that children with strong object control may find it easier to transfer their body awareness from land to water, thus mastering kicking and arm-stroking techniques more quickly. Good object control enables children to perform aquatic movements more efficiently, reducing unnecessary energy expenditure and achieving greater progress within limited training time (35). When faced with different aquatic situations (such as varying water currents and postures), children with strong object control can adjust more flexibly and adapt to changes. Similarly, the study by Lauren et al. pointed out that swimming interventions can significantly improve children’s motor skills, including balance ability and object control skills, and compared to other sports, swimming shows a more significant effect in promoting the development of FMS (25).

Before the intervention, there was no significant correlation between FMS and the pre-test of AC, possibly because all children had no swimming learning experience, and their AC was at a basic level. The advantages of FMS may not have been effectively transferred and applied to the water, or the children may not have had the opportunity to demonstrate or develop this migration ability. Merely having good FMS does not mean that children can naturally perform well in the water. The advantages of FMS need to be transformed into water ability through specific training under professional guidance. This suggests that in early childhood development, focusing on and enhancing their FMS, especially the ability to control objects, may have a long-term positive impact on their future participation in AC. In addition, this study did not systematically control for children’s participation in physical activities outside of the intervention or other exercise-based activities, which may affect the interpretation of the relationship between fundamental motor skills and aquatic abilities. At the same time, we will expand the description in the limitations section, emphasizing that these variables need to be controlled in future research through questionnaire surveys or longitudinal designs.

The conclusions of this study can directly guide the practice of aquatic education. When designing aquatic motor skills courses for preschool children, training sessions aimed at improving children’s body control abilities should be consciously integrated. For children with a weak foundation in Fundamental Movement Skills (FMS), it may be considered to conduct FMS training on land before they engage in aquatic activities, thereby laying a foundation for them to better adapt to and learn aquatic skills. In the future, when assessing children’s AC, some FMS indicators (especially those related to object control) can also be included in the assessment system to gain a more comprehensive understanding of children’s AC.

This study still has some limitations. First, this study only selected 120 preschool children from Beijing, and the sample size is relatively limited, which may not fully represent the FMS and aquatic competence levels of preschool children in different regions and cultural backgrounds across the country. Future research can consider expanding the sample size and including samples from more diverse geographical and cultural backgrounds. Second, the 6-week intervention period may be insufficient for a complete assessment of the long-term impact of FMS on AC. Although the study showed significant short-term effects, long-term follow-up studies could explore more complex and stable correlations that may emerge between FMS and the acquisition of AC as the learning deepens. Finally, although we controlled for gender and age (through stratified randomization), we did not control for other potential influencing factors such as children’s family sports exercise habits and parents’ attitudes toward children’s sports participation. However, it is crucial to incorporate these factors into the design of future research. Future research can collect and control these variables through methods such as questionnaire surveys or longitudinal designs to more clearly explain the relationship between FMS and AC more clearly.

The conclusion of this study is that for preschool children aged 4–6, boys’ fundamental motor skills, especially object control abilities, are positively correlated with their acquisition of AC. Systematic learning programs for aquatic motor skills can not only significantly enhance children’s AC but also promote the development of their fundamental motor skills. Children with strong body control show more significant improvements in their AC after receiving professional training. The findings of this study provide valuable references for the theory and practice of physical education for preschool children, especially in aquatic education, emphasizing the necessity of comprehensively cultivating children’s fundamental motor skills in the early stages, laying a solid foundation for their future development in aquatic and other sports fields.

## Data Availability

The raw data supporting the conclusions of this article will be made available by the authors, without undue reservation.
